# Decoding Time-Varying Functional Connectivity Networks via Linear Graph Embedding Methods

**DOI:** 10.3389/fncom.2017.00014

**Published:** 2017-03-20

**Authors:** Ricardo P. Monti, Romy Lorenz, Peter Hellyer, Robert Leech, Christoforos Anagnostopoulos, Giovanni Montana

**Affiliations:** ^1^Department of Mathematics, Imperial College LondonLondon, UK; ^2^Computational, Cognitive and Clinical Neuroimaging Laboratory, Imperial College London, The Hammersmith HospitalLondon, UK; ^3^Department of Bioengineering, Imperial College LondonLondon, UK; ^4^Center for Neuroimaging Sciences, Institute of Psychiatry, Psychology and Neuroscience, King's College LondonLondon, UK; ^5^Department of Biomedical Engineering, King's College London, St Thomas' HospitalLondon, UK

**Keywords:** dynamic networks, graph embedding, functional connectivity, brain decoding, visualization

## Abstract

An exciting avenue of neuroscientific research involves quantifying the time-varying properties of functional connectivity networks. As a result, many methods have been proposed to estimate the dynamic properties of such networks. However, one of the challenges associated with such methods involves the interpretation and visualization of high-dimensional, dynamic networks. In this work, we employ graph embedding algorithms to provide low-dimensional vector representations of networks, thus facilitating traditional objectives such as visualization, interpretation and classification. We focus on linear graph embedding methods based on principal component analysis and regularized linear discriminant analysis. The proposed graph embedding methods are validated through a series of simulations and applied to fMRI data from the Human Connectome Project.

## 1. Introduction

Functional connectivity describes the pairwise statistical dependencies which exist across spatially remote brain regions (Friston, 2011). When studied across multiple regions, connectivity is often represented as a network or a graph (Bullmore and Sporns, 2009). Until recently, many studies had implicitly assumed that the statistical dependencies across spatially remote brain regions remained constant, implying that the associated network did not vary. In such a setting, a single network is sufficient to describe the functional relationships across regions. However, there is growing evidence to suggest that fMRI data displays non-stationary properties (Hutchinson et al., 2013), indicating that the associated functional connectivity networks may vary over time. This is particularly the case in the context of task-based studies (Calhoun et al., 2014).

This has led to the development of several methods through which to quantify the dynamic properties of functional connectivity networks (Allen et al., 2012; Leonardi et al., 2013; Monti et al., 2014). Such methodologies have provided insights relating to the dynamic restructuring and temporal evolution of the human connectome and may potentially provide insight relating to various neurological and psychiatric conditions (Damaraju et al., 2014; Demirtaş et al., 2016; Sourty et al., 2016).

However, obtaining robust and easily interpretable insights from the results of such algorithms raises important statistical challenges. The difficulties are further exacerbated by the fact that often a distinct network is estimated at each observation and potentially across many subjects. One potential solution involves testing for statistical correlations between the estimated edge strengths over time and underlying changes in cognitive task, thereby reporting the set of edges which is functionally modulated by a given task. While such methods are often advocated (Yao et al., 2016; Monti et al., 2017), they effectively study each edge independently thereby failing to account for the structured nature of networks. Crucially, by studying edges on an individual basis such methods fundamentally ignore the notion that the brain is a functionally connected network (Sporns et al., 2004; Bressler and Menon, 2010). A related approach involves the use of clustering methods such as *k*-means (Allen et al., 2012). Such methods are able to identify *state* networks which can capture the current connectivity structure at specific points in time. However, clustering based methods require the definition of a distance metric which is difficult to define in the context of graphs (Richiardi et al., 2010). Finally, time-varying graph metrics may also be employed (Calhoun et al., 2014), where metrics such as the degree or betweenness centrality are tracked over time. However, it if often difficult *a priori* to know which metrics to consider and there is no guarantee that predefined metrics will necessarily capture all the relevant changes in connectivity structure.

In this work, we look to address the challenges associated with interpreting time-varying, high-dimensional networks via the use of linear graph embedding methods. Generally speaking, the objective of graph embedding techniques is to map estimated graphs into a (potentially low-dimensional) vector space (Yan et al., 2007). This facilitates tasks such as visualization and classification by translating the problem from the graph domain into a Euclidean space, where traditional classification and visualization techniques can be readily applied.

While a wide range of graph embedding techniques may be employed, in this work we limit ourselves to consider only methods based on linear projections over the edge structure of an estimated graph. This allows us to obtain a clear interpretation of the embedding in the context of functional connectivity. As a result, we consider two distinct graph embedding algorithms. The first embedding considered is based on Principal Component Analysis (PCA). This embedding, which is closely related to the work of Leonardi et al. (2013), can be interpreted as mapping graphs into a low-dimensional vector space that captures the maximal variability in the estimated functional connectivity networks over time. The objective of PCA is to recover orthogonal projections of the data which best explain the variability present (Jolliffe, 2002). In this manner, PCA is able to reduce the dimensionality of data where there exists a large number of interrelated variables. Due to the unsupervised nature of this embedding, it is ideally suited for the study of both resting-state as well as task-based fMRI data. The second approach is based on regularized Linear Discriminant Analysis (LDA). This method serves to recover a low-dimensional embedding that maximizes the discriminatory power across various tasks or states. The supervised nature of such an embedding is particularly suitable for task-based experiments, where changes in cognitive task are known and the objective is to recover the associated changes in the connectivity structure.

The remainder of this manuscript is organized as follows: We introduce the aforementioned linear graph embedding techniques based on principal component and linear discriminant analysis in Section 2. An extensive simulation study is presented in Section 3. Finally, in Section 4 the proposed methods are applied to task-based fMRI datasets taken from the the Human Connectome Project (Elam and Van Essen, 2014).

## 2. Methods

Throughout this section it is assumed that estimates of time-varying functional connectivity networks have been obtained across a cohort of *S* subjects. We write $\Theta_{i}^{\left(s\right)}\in {\mathbb{R}}^{p\times p}$ to denote the estimated functional connectivity network for the *s*th subject at the *i*th observation. Each $\Theta_{i}^{\left(s\right)}$ therefore captures the statistical dependencies across *p* regions of interest (ROIs) at the *i*th observation. Throughout this work it is assumed that $\Theta_{i}^{\left(s\right)}$ is a sparse estimate of the inverse covariance matrix, thereby encoding the conditional dependence (i.e., partial correlation) structure across nodes. As such, the (*j, k*) element of $\Theta_{i}^{\left(s\right)}$ captures the partial correlation between the *j*th and *k*th regions. Furthermore, any pair of regions are conditionally independent if and only if the corresponding entry of $\Theta_{i}^{\left(s\right)}$ is zero (Lauritzen, 1996). We note that the proposed methods are also applicable in the context of alternative network estimation methods (see Smith et al., 2011 for an extensive review).

The dynamic properties of functional connectivity networks can be quantified in many ways. One popular method for estimating such networks involves the use of sliding windows (Hutchinson et al., 2013). Here observations lying within a time window of fixed length are used to calculate the functional connectivity at a given time. The window is subsequently shifted, allowing for the estimating of dynamic networks. Alternative methods, based on approaches such as change-point detection (Cribben et al., 2012; Gibberd and Nelson, 2014) and forgetting factors have also been proposed (Monti et al., 2017).

In this work our objective is to understand dynamic functional connectivity networks using linear graph embedding methods. Such methods allow for the representation of graphs or networks in real-valued vector spaces, resulting in two advantages. First, by embedding graphs in a Euclidean vector spaces we are able to employ traditional visualization and classification techniques. Second, by focusing on linear projections we are able to directly interpret the embeddings in the context of functional connectivity networks. The linear embedding methods considered in this work are based on principal component analysis and regularized linear discriminant analysis. Such methods correspond to unsupervised and supervised learning algorithms, respectively, indicating the they may be used in conjunction to further understand dynamic connectivity networks.

The remainder of this section is organized as follows: we introduce and discuss graph Laplacians in Section 2.1. In Sections 2.2 and 2.3 we introduce two distinct graph embedding methods.

### 2.1. Graph laplacians

The graph embedding techniques described in this work are based on the Laplacian of each estimated functional connectivity network. While there are a wide variety of different graph Laplacians which may be employed, throughout this work we consider the normalized graph Laplacian (Chung, 1997). This is formally defined as:

(1) $$\begin{array}{rc} L_{i}^{\left(s\right)} & ={\left(D_{i}^{\left(s\right)}\right)}^{-\frac{1}{2}}\left(D_{i}^{\left(s\right)}-\Theta_{i}^{\left(s\right)}\right){\left(D_{i}^{\left(s\right)}\right)}^{-\frac{1}{2}} \end{array}$$

(2) $$\begin{array}{r} =I-{\left(D_{i}^{\left(s\right)}\right)}^{-\frac{1}{2}}\Theta_{i}^{\left(s\right)}{\left(D_{i}^{\left(s\right)}\right)}^{-\frac{1}{2}}, \end{array}$$

where we define $D_{i}^{\left(s\right)}=\text{diag}\left(\Theta^{\left(s\right)}\right)$ and *I* to be the identity matrix. The benefit of employing the normalized graph Laplacian is that each matrix $L_{i}^{\left(s\right)}$ summarizes the partial correlations across all regions. This can be seen by noting that the (*j, k*) element of $L_{i}^{\left(s\right)}$ is formally defined as:

(3) $$\begin{array}{l} {\left(L_{i}^{\left(s\right)}\right)}_{j,k}=\left\{ \begin{array}{cc} 0\; & \text{if}j=k\\ -\frac{{\left(\Theta_{i}^{\left(s\right)}\right)}_{j,k}}{{\left({\left(\Theta_{i}^{\left(s\right)}\right)}_{j,j}{\left(\Theta_{i}^{\left(s\right)}\right)}_{k,k}\right)}^{\frac{1}{2}}}\; & \text{if}j\neq k \end{array}\right. \end{array}$$

Furthermore, it is clear from Equation (3) that such a transformation serves to normalize the estimated edge weights by the variance of each node. We note that due to the symmetric nature of the Laplacian matrix, it is fully characterized by its upper-triangular entries. We define the set of Laplacian matrices for a given subject to be $L^{\left(s\right)}=\left\{L_{i}^{\left(s\right)}:i=1\ldots ,n\right\}$. In the remaining sections, we employ *L*^(*s*)^ directly as input to the proposed graph embedding algorithms. Moreover, we define vec(L(s))∈ℝn×(p2) to be a matrix where the *i* row corresponds to the vectorized upper-triangular entries of the normalized Laplacian at the *i*th observation. The matrix, *L*, consisting of all vectorized Laplacians across all subjects can subsequently be defined as:

(4) L=[vec(L(1))T,…vec(L(S))T]T∈ℝS·n×(p2).

**Figure 1:**
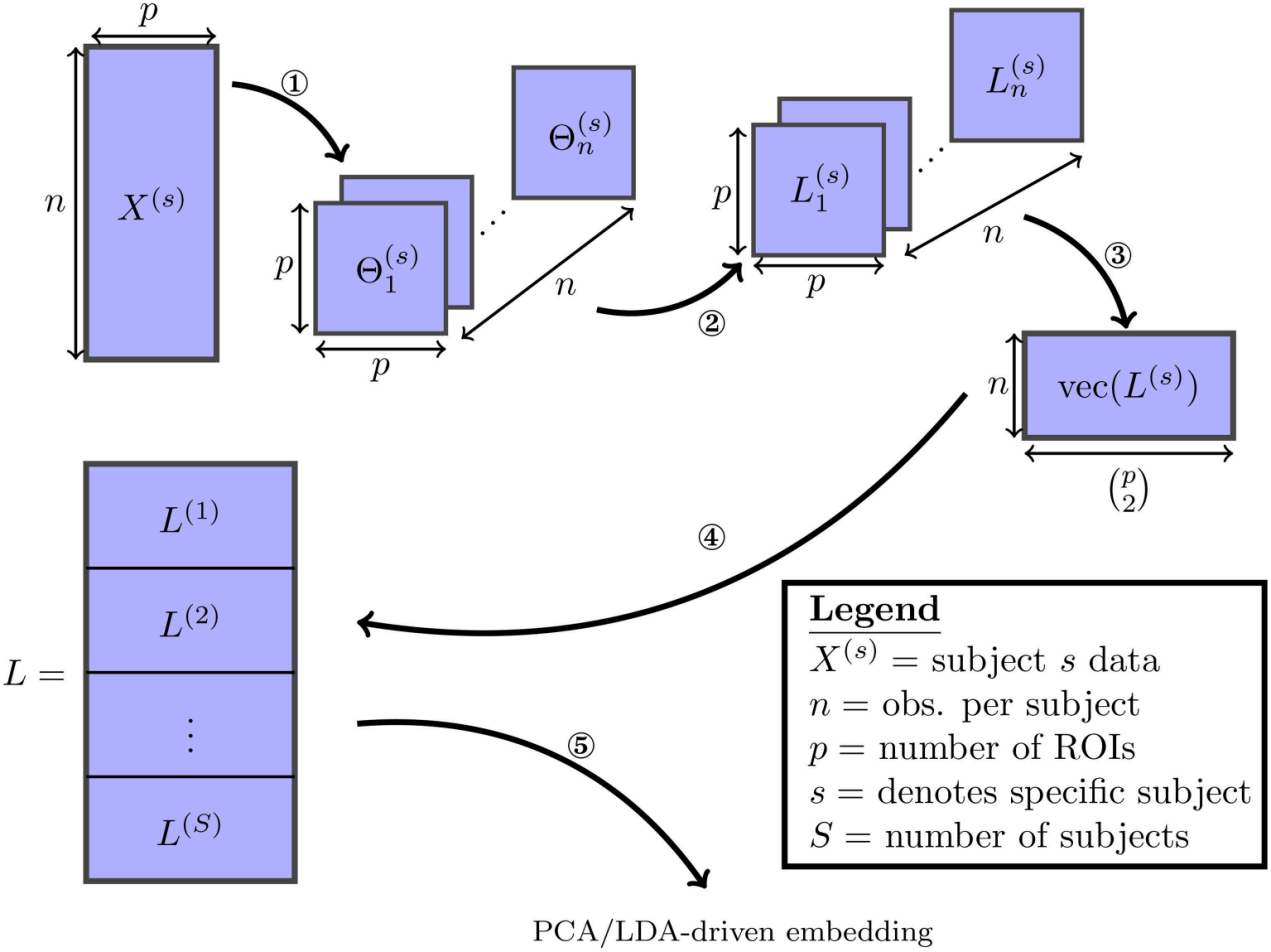
**The various steps involved in the proposed embedding method are visualized: (1) the SINGLE algorithm detailed in Appendix A (Supplementary Material] is used to obtain estimates of time-varying precision matrices**. (2) The precision matrices are transformed to Laplacian matrices. (3) The Laplacian matrices are vectorized by taking their upper-triangular components. (4) The vectorized Laplacians of all subjects are stacked vertically. (5) Finally the PCA/LDA-driven embeddings are estimated.

This process is described in Figure 1. It follows that each column of *L* corresponds directly to one of the (p2) possible edges. As both embeddings studied here consist of linear projections of *L* onto lower-dimensional subspaces, they can each be understood as a a linear combination over the set of edges in a functional connectivity network.

### 2.2. Unsupervised PCA-driven embedding

In this section we discuss an unsupervised embedding method through which to obtain a low-dimensional embedding that maximizes the amount of explained variance. Following from the method described in Leonardi et al. (2013), we look to achieve this by applying Principal Component Analysis (PCA) to *L*. This will yield the linear combination of edges that best summarize the variability over estimated functional connectivity networks over time.

Formally, PCA is an unsupervised dimensionality reduction technique which produces a new set of uncorrelated variables. This is achieved by considering the *k* leading eigenvectors of the covariance matrix *L*^*T*^*L*, defined as the principal components Pk∈ℝk×(p2). The principal components, *P*_*k*_, can be studied in two ways. First, by considering the entries of each principal component we are able to quantify the contribution of each edge to the principal component in question. As such, combinations of edges which co-vary highly within a dataset can therefore be expected to provide a large contribution to the leading principal components. As each principal component is defined as a weighted sum over the set of edges, they may be interpreted as a recovering a functional connectivity network. Second, the embedding produced by *P*_*k*_ is obtained as:

(5) $$\begin{array}{l} P_{k}\cdot \text{vec}\left(L^{\left(s\right)}\right)\in {\mathbb{R}}^{k\times n}. \end{array}$$

This yields a *k*-dimensional graph embedding for each subject at each of the *n* observations. This serves as a low-dimensional representation networks which can be employed in tasks such as classification or visualization.

### 2.3. Supervised LDA-driven embedding

While the PCA-driven embedding detailed in Section 2.2 was motivated by understanding the components of functional connectivity which demonstrated the greatest variability over time, we may also be interested in understanding which functional networks are most discriminative across multiple tasks. In this section we describe a supervised graph embedding methods through which to achieve this goal.

We propose the use of LDA to learn the functional connectivity networks which are most discriminative between tasks. LDA is a simple and robust classification algorithm which can also be interpreted as a linear projection (Hand, 2006). As a result, LDA reports the linear combination of edges which are most discriminative between tasks. These can subsequently be interpreted as a discriminative embedding which reports changes in functional connectivity induced by a given task.

In high-dimensional supervised learning problems, such as the one considered in this work, it is of paramount importance to avoid overfitting. Two popular methods to guard against overfitting involve the introduction of regularization, thereby penalizing overly complex models which are more susceptible to overfitting, and cross-validation. Here a combination of both approaches is employed. The proposed graph embedding first employs ℓ_1_ regularization methods in order to reduce the number of candidate edges to be included. This acts as a variable screening procedure whose goal is to retain all discriminative variables (in our case edges) whilst removing noise variables. The latter will correspond to edges which are not discriminative of the tasks in question. Such a variable screening procedure is discussed in Section 2.3.1. Given a subset of screened variables, an LDA classifier is subsequently trained as described in Section 2.3.2.

#### 2.3.1. Variable screening

In this section we detail the variable screening procedure employed within the LDA-driven embedding. As discussed previously, overfitting is a significant problem in the context of high-dimensional supervised learning. Throughout this work, we look to address this issue via the use of a screening procedure.

Formally, the objective of the screening procedure employed is to significantly reduce the number of candidate variables to p′<<(p2). This is achieved by retaining only the most reproducible edges and discarding all others. As such, an independent ℓ_1_ penalized LDA classifier was estimated for each subject. Such models can be efficiently estimated as described in Clemmensen et al. (2011) and provide the additional benefit of performing variable selection. The severity of the ℓ_1_ penalty is parameterized by a regularization parameter. Such a parameter plays a fundamental role in the variable selection procedure and must therefore be carefully selected. While there are a wide range of methods through which to select the regularization parameter, in this work cross-validation was employed.

As a result, a regularized LDA model was estimated for each subject. This resulted in a sparse discriminant vector, β^(*s*)^, for each subject. The sparse support of each β^(*s*)^ may then be studied in order to recover the set of most reproducible edges. Formally, we define the reproducibility of the *i*th edge to be:

(6) $$\begin{array}{l} \eta_{i}=\frac{1}{S}\overset{S}{\underset{s\;=\;1}{\sum }}𝟙\left(\beta_{i}^{\left(s\right)}\neq 0\right). \end{array}$$

Thus η_*i*_ effectively counts the proportion of subjects in which the *i*th edge is retained within a regularized LDA classifier. It follows that edges may therefore be ranked according to their reproducibility across a cohort of subjects. The proposed screening procedure retains all edges where η_*i*_ is greater than some specified threshold, ρ ∈ [0, 1]. This serves to retain only the edges which are active within at least ρ% of all subjects. The set of screened edges which are active is defined as:

(7) $$\begin{array}{l} A=\left\{i:\eta_{i}>\rho \right\}. \end{array}$$

Such a screening procedure can be interpreted as performing stability selection, as described in Meinshausen and Bühlmann (2010), where the sub-sampling is performed by studying each subject independently. This serves to discard a large number of noisy and non-informative variables, yielding a Laplacian matrix, *L*′ ∈ ℝ^*S*·*n*×|*A*|^, consisting of only selected variables which have demonstrated reproducible discriminative power across all subjects. Finally, we note that it is important that the aforementioned variable selection procedure is implemented using only the training data and not the test dataset.

#### 2.3.2. LDA embedding

The first step of the proposed LDA-driven embedding corresponds to splitting the data into training and test data. Throughout this work, the training and test data were obtained by randomly dividing subjects. As such, a subset of subjects, *S*_*train*_, were considered for training and the remainder, *S*_*test*_, were retained for the purpose of testing. The variable selection procedure, detailed in Section 2.3.1, is subsequently applied to data corresponding to training subjects in order to prune the number of edges considered.

Once the variable screening procedure has been employed, screened Laplacian matrices are obtained for the training and test data, respectively. We write $L_{train}^{\prime }$ and $L_{test}^{\prime }$ to denote the training and test screened Laplacian matrices, respectively. An LDA classifier is subsequently training using only $L_{train}^{\prime }$. The performance of the classifier is validated by studying $L_{test}^{\prime }$, which serves to provides important metrics about the reproducibility of the embedding and its generalization performance. The full procedure to fit the LDA-driven embedding is described in Algorithm 1.

**Algorithm 1:**
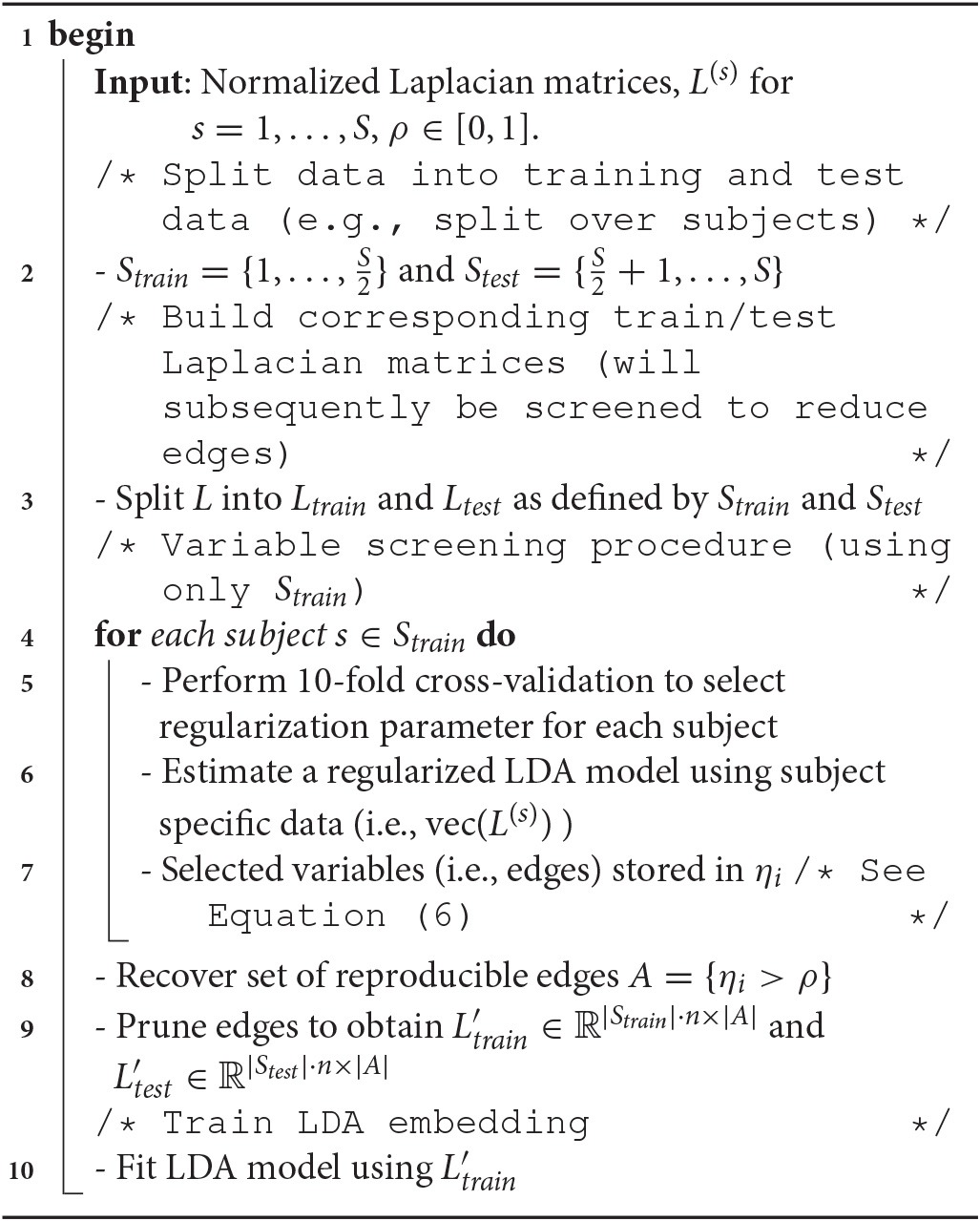
Sparse LDA-driven embedding

### Software

Python code implementing all the graph embedding algorithms discussed in this section are provided in the supplementary material or can be directly downloaded from https://github.com/piomonti/pyLGE.

## 3. Simulation study

In this section we provide empirical evidence to demonstrate the capabilities of the two graph embeddings methods introduced in Section 2. Throughout these simulations, we produce simulated time series data giving rise to a number of connectivity patterns which reflect those reported in real fMRI data. The data is generated such that the underlying connectivity varies over time and the Smooth Incremental Graphical Lasso Estimation (SINGLE) algorithm (Monti et al., 2014) is subsequently employed to obtain estimates of time-varying connectivity networks. A detailed review of the SINGLE algorithm is provided in Appendix A (Supplementary Material). While the SINGLE algorithm was employed in this work, it follows that any alternative algorithm could also have been used. The objective of this simulation is therefore to quantify how reliably the proposed graph embedding algorithms are able to capture changes in connectivity structure.

### 3.1. Simulation settings

In order to thoroughly test the capabilities of the proposed graph embedding algorithms, we look to generate simulated data which contains many of the characteristic properties often associated with fMRI data. There are two main properties of fMRI data which we wish to recreate in the simulation study. The first is the high autocorrelation which is typically present in fMRI data (Poldrack et al., 2011). The second property we wish to recreate is the topological properties of the simulated functional functional connectivity networks. While there is an extensive literature studying such properties, we look to generate synthetic data were the covariance structure demonstrates either small-world or scale-free topologies. This is motivated by research which suggests functional connectivity networks display thees topological properties (Bullmore and Sporns, 2009) as well as the fact that such networks can be easily simulated in practice.

In order to achieve this, we follow the simulation study described (Monti et al., 2014). This involved the use of vector autoregressive (VAR) processes to generate autocorrelated, multivariate time-series. Briefly, VAR models serve as a generalization of univariate autoregressive models and are often employed to capture interdependencies between multiple time series (Cribben et al., 2012). The use of VAR models therefore allowed for the encoding of both autocorrelations within components as well as cross-correlations across nodes. Formally, the the covariance structure across various nodes was simulated using a random graph algorithm (e.g., an Erdős-Rényi random graphs) and the use of VAR models served to introduce autocorrelation between successive observations as would be expected in the context of fMRI data. We validate the performance of each graph embedding method using three distinct random graph algorithms: Erdős-Rényi random graphs (Erdos and Renyi, 1959), scale-free random graphs obtained by using the preferential attachment model of Barabási and Albert (1999) and small-world random graphs obtained using the Watts and Strogatz (1998) model. Erdős-Rényi random graphs are included as they correspond to the simplest and most widely studied type of random network while the use of scale-free and small-world networks is motivated by the fact that they are each known to each resemble different aspects of brain connectivity networks.

Following Monti et al. (2014), we fix the edge strength between nodes to be 0.6 in the case of Erdős-Rényi random networks. In the case of the scale-free and small-world networks we randomly sample the edge strengths uniformly from [−½, −¼]∪[¼, ½] This choice was motivated by recent results which suggest the edges in functional connectivity networks are highly heterogeneous (Markov et al., 2013). In this manner the performance of the proposed methods was studied in the context of three distinct settings. The first setting corresponds to Erdős-Rényi random networks where the edge weights remain fixed and is arguably the easiest setting. In the second and third settings, more complex random graph algorithms are employed and the edge weights are allowed to vary, thereby increasing the difficulty of the associated task. 6 We note that the density of the simulated networks was fixed such that 20% of all possible edges were present. Finally, we note that within each simulation the network structure was shared across all subjects, however, the data for each subject was randomly generated. We note that this is just one of a range of potential simulation settings which may be employed but which serve to empirically validate the proposed method.

In many task based studies, subjects are required to alternate between performing a cognitive task and resting in a cyclic fashion. As such, the simulations presented in this work consist of a cyclic connectivity structure, where the underlying connectivity varies between two simulated networks. As a result, multivariate, simulated data was generated where the underlying covariance structure alternated in a cyclic fashion. As noted previously, we consider three distinct network structures: Erdős-Rényi, scale-free and small-world networks. Furthermore, networks were simulated with *p* ∈ {10, 25, 50, 100, 150} nodes, respectively, while the number of observations within each segment remained fixed at *n* = 100. This allows for the study of the behavior the proposed of graph embedding techniques as the ratio ^*n*^/_*p*_ decreases. Throughout this simulation, *S* = 20 datasets where independently simulated as described above. This served to replicate a typical fMRI study, where data is typically collected for a group of around 20 subjects.

### 3.2. Performance metrics

In order to evaluate the empirical performance of the graph embedding methods we consider the discriminatory power of the estimated embeddings when predicting the underlying covariance structure. As the underlying covariance structure is simulated to alternate between two network structures, this corresponds to binary classification task and traditional classification scores, such as the area under the ROC curve (AUC), can be employed (Krzanowski and Hand, 2009). The benefit of employing the AUC score to quantify performance of each graph embedding is that no additional classification algorithm or threshold is required. This is because the AUC measure effectively computes the discriminative capability of each graph embedding at all possible thresholds. More concretely, the embedding scores corresponding to either the leading principal component of discriminant scores can be studied directly in this manner. Finally, we note that throughout the simulation study presented in this work only the leading embeddings were considered. This serves to demonstrate that the proposed methods are able to detect significant changes in the covariance structure. However, it may also be of interested to consider the detection of more subtle changes. Theoretically, such changes would be reported in higher level embeddings such as the second or third principal component embeddings, however we do not study these properties within this simulation study.

### 3.3. Results

Data was simulated as described in Section 3.1. The SINGLE algorithm was subsequently applied in order to estimate time-varying functional connectivity networks for each subject. A detailed review of the algorithm is provided in Appendix A (Supplementary Material). The application of the SINGLE algorithm required the specification of three hyper-parameters which were selected as follows: the kernel width parameter was estimated once across all subjects using cross-validation. The remaining regularization parameters were selected as detailed in Monti et al. (2014): this involved minimizing the Akaike Information Criterion (AIC) on a subject-by-subject basis. Given the estimated networks, the two graph embedding methods introduced in Section 2 were applied. Half of the *S* = 20 subjects were selected as a training sample, and the networks for the remaining subjects were kept as a validation set.

#### 3.3.1. PCA-driven embeddings

**Figure 2:**
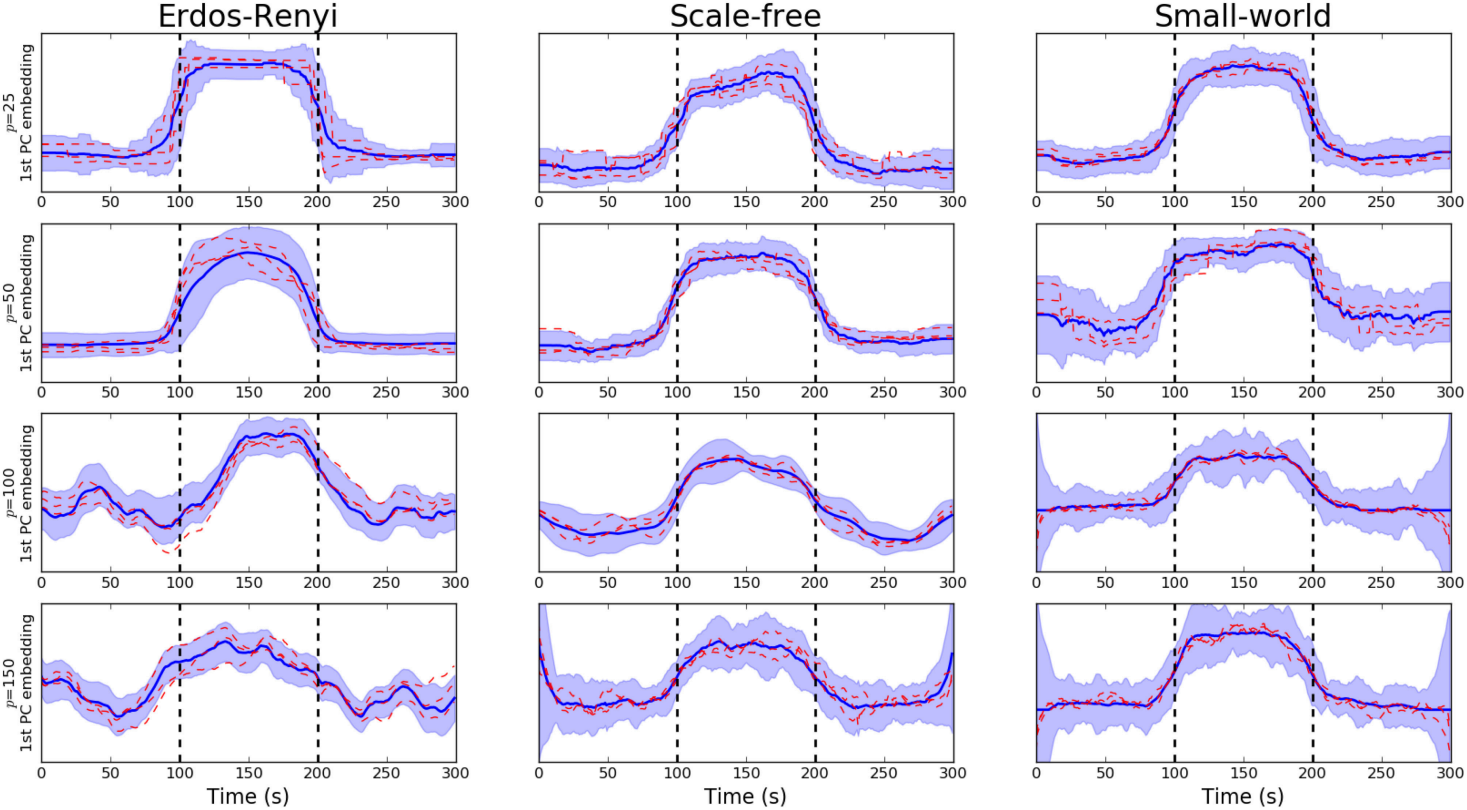
**Visualization of PCA-driven embeddings for simulated data where the number of nodes varies ***p*** ∈ {20, 50, 100, 150}**. Each panel shows the mean PCA-driven embedding over 10 unseen, simulated datasets (i.e., 10 unseen subjects). The thick blue line corresponds to the average of the leading principal component across subjects, defined in Equation (5). Standard deviations are indicated by the shaded regions. The dashed red lines show the leading principal component for three randomly selected subjects, thereby providing an indicating of the variability across subjects. Results are shown when the underlying connectivity structure was simulated using three distinct graph algorithms: Erdős-Rényi, scale-free and small-world random graphs. Vertical dashed lines indicate a change in covariance structure.

We begin by studying the performance of the PCA-driven embeddings. Recall that the objective of this method is to obtain a low-dimensional embedding which maximizes the amount of explained variance. Figure 2 provides an initial flavor for the capabilities of the proposed method where the embedding based on the leading principal component has been visualized over unseen subjects for various different values of *p*. Recall that the underlying covariance structure has been simulated in a cyclic fashion such that the first and third segments share the same connectivity structure. Change points are denoted by dashed, vertical lines. The results presented in Figure 2 serve to demonstrate the performance of the PCA-driven embedding as the number of regions *p* increases. In particular, we note that as *p* increases, the performance of the proposed method deteriorates. This is to be expected as the increases in the number of regions, *p*, increases the difficulty associated with the estimation of the underlying covariance matrices. The dashed red lines in Figure 2 correspond to the graph embeddings obtain for specific unseen subjects, thereby demonstrating that the estimated embeddings are robust.

In order to be obtain a more comprehensive understanding regarding the performance of the embedding, we consider the predictive power of the embeddings when trying to uncover the underlying covariance structure. In this setting, the underlying covariance structure was treated as a binary variable with two classes: each of which serves to indicate one of the two underlying connectivity regimes. The embedding corresponding to the leading principal component was then employed to discriminate across the class. The AUC score was then employed to obtain a measure of the discriminative capabilities of the embedding (Krzanowski and Hand, 2009). Detailed results are provided in Table 1A where the mean AUC score across all unseen simulated subjects is reported together with the standard deviation. As expected, there is a clear decline in the discriminative capabilities of the embedding as the dimensionality of the network increases.

**Table 1:** **Mean AUC scores for each of the proposed graph embeddings are shown when the underlying covariance structure is simulated using three distinct methods**.

***p***	**Erdős-Rényi**	**Scale-free**	**Small-world**
**(A) PCA-DRIVEN**			
10	0.94 (0.02)	0.94 (0.04)	0.92 (0.05)
25	0.95 (0.03)	0.88 (0.07)	0.80 (0.08)
50	0.91 (0.03)	0.84 (0.06)	0.79 (0.07)
100	0.73 (0.05)	0.76 (0.06)	0.70 (0.05)
150	0.66 (0.06)	0.64 (0.05)	0.67 (0.06)
**(B) LDA-DRIVEN**			
10	0.97 (0.01)	0.96 (0.05)	0.97 (0.06)
25	0.95 (0.03)	0.93 (0.06)	0.83 (0.07)
50	0.90 (0.04)	0.89 (0.06)	0.78 (0.07)
100	0.75 (0.06)	0.77 (0.05)	0.73 (0.06)
150	0.68 (0.05)	0.70 (0.04)	0.69 (0.06)

*Results are presented for networks with varying numbers of nodes, p. Standard deviation are provided in brackets*.

#### 3.3.2. LDA-driven embeddings

While the PCA-driven embeddings are motivated by the need to understand components of estimated networks which demonstrate the greatest variability, it is also important to consider embeddings which are discriminative across multiple cognitive tasks. The LDA-driven embeddings introduced in Section 2.3 are one potential method through which to achieve this. Briefly, the objective of such an embedding is to learn a linear combination of edges which is maximally discriminative across across tasks.

The fundamental difference between the PCA and LDA-driven embeddings is that the latter is a supervised embedding. As a result, it is crucial to avoid any potential overfitting. As described in Section 2.3, the proposed method employs a variable screening procedure based on regularized models. This use of regularization also serves to penalize complex models which are naturally more prone to overfit.

**Figure 3:**
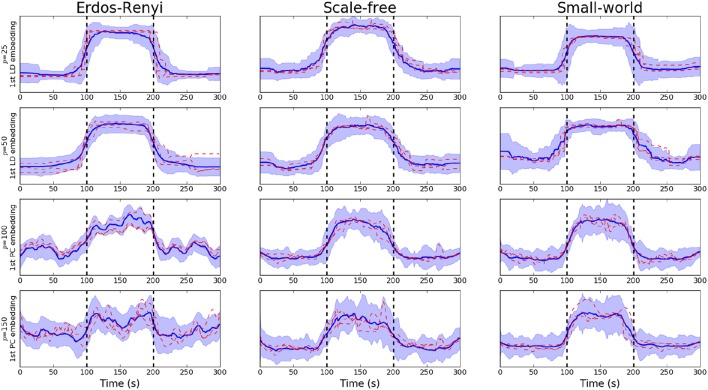
**Visualization of LDA-driven embeddings for simulated data where the number of nodes varies ***p*** ∈ {20, 50, 100, 150}**. Each panel shows the mean LDA-driven embedding over 10 unseen, simulated datasets (i.e., 10 unseen subjects). The thick blue line corresponds to the average of the linear discriminant scores across subjects. Standard deviations are indicated by the shaded regions. The dashed red lines show the linear discriminant scores for three randomly selected subjects, thereby providing an indicating of the variability across subjects. Results are shown when the underlying connectivity structure was simulated using three distinct graph algorithms: Erdős-Rényi, scale-free and small-world random graphs. Vertical dashed lines indicate a change in covariance structure.

We note that the underlying covariance structure was simulated in a cyclic fashion which alternated between two distinct regimes. As a result, the objective of the proposed embedding is to differentiate between two distinct classes. Due to the properties of LDA, this results in a 1-dimensional embedding (Hastie et al., 2009). This embedding is visualized for networks of varying dimensions in Figure 3. Following the discussion in Section 3.3.1, we notice there is a clear deterioration in the quality of the embeddings recovered as the dimensionality, *p*, increases. This is to be expected as the number of observations, *n*, remains fixed. More comprehensive results are provided in Table 1B, where the mean AUC score over unseen datasets is reported. As with the PCA-driven embeddings, we note there is a drop in performance as the number of nodes, *p*, increases. We note that the LDA-driven embeddings typically out-perform the PCA-driven embeddings. We attribute this to the supervised nature of the LDA-driven embedding. Formally, the objective of PCA-driven embedding is to learn a low-dimensional representation which captures maximal variance. A decrease in the ratio ^*n*^/_*p*_ leads to a corresponding increase in the variability of estimated networks. This may be partially responsible for the difference in embeddings shown in Figure 2 as *p* increases. On the other hand, the objective of the proposed LDA-driven embedding is to learn a linear combination of edges which is discriminative across multiple classes. As such, the drop in the ratio ^*n*^/_*p*_ does not result in significant changes to the magnitude of estimated embeddings.

## 4. Application

In this section we present an application of the proposed graph embedding techniques to a task-based fMRI dataset taken from the Human Connectome Project (Elam and Van Essen, 2014).

### 4.1. HCP working memory data

The data consisted of working memory task data taken from the Human Connectome Project (Elam and Van Essen, 2014). During the tasks subjects were presented with blocks of trials consisting of either 0-back or 2-back working memory tasks. Two datasets were provided for each subject, corresponding to a left-right (LR) and right-left (RL) acquisitions. Throughout both the left and right acquisition subjects were required to perform the same working memory task with the only difference that the block design of the task varied slightly from one acquisition to another. Throughout this work, they were treated as separate scans and studied independently. Data corresponding to *S* = 206 of the possible 500 subjects was selected at random^1^. Thus a total of 2 × 206 = 412 datasets were studied.

#### 4.1.1. Data pre-processing

Preprocessing involved regression of Friston's 24 motion parameters from the fMRI data (Friston et al., 1996). Sixty-eight cortical and sixteen subcortical ROIs were derived from the Desikan-Killiany atlas (Desikan et al., 2006) and the ASEG atlas (Fischl et al., 2002), respectively. A full list of the regions employed, together with their MNI coordinates, is provided in Appendix B (Supplementary Material). Mean BOLD timeseries for each of these 84 ROIs were extracted and further cleaned by regressing out timeseries sampled from white matter and cerebrospinal fluid. Finally, the extracted timecourses were high-pass filtering using a cut-off frequency of $\frac{1}{150}$ Hz.

#### 4.1.2. Network estimation

As in the simulation study, time-varying functional connectivity networks were estimated for each subject using the SINGLE algorithm. This required the specification of three hyper-parameters: the width, *h*, of the Gaussian kernel as well as the regularization parameters, λ_1_ and λ_2_. A fixed kernel width of *h* = 15, selected via cross-validation, was employed across all subjects. The regularization parameter were selected on a subject-by-subject basis by minimizing AIC. This involved an extensive grid-search over all possible combinations of λ_1_ and λ_2_. In order to reduce the computational burden associated with selecting λ_1_ and λ_2_, an initial search was performed on a reduced subset of the subjects. This served to identify a region of the parameter space that was consistently selected across subjects, thereby greatly reducing the computational cost.

#### 4.1.3. Results

The estimated functional connectivity networks produced by the SINGLE algorithm were subsequently analyzed using the proposed graph embedding methods. Recall that the objective of the PCA-driven embedding was to provide a low-dimensional embedding which captures a large portion of the variability present in the data. This was achieved in an unsupervised manner by considering the embeddings associated with the *k* = 2 reading principal components. We note that both the LR and RL acquisitions for each subject where considered simultaneously as the goal was to understand variability across the entire population.

**Figure 4:**
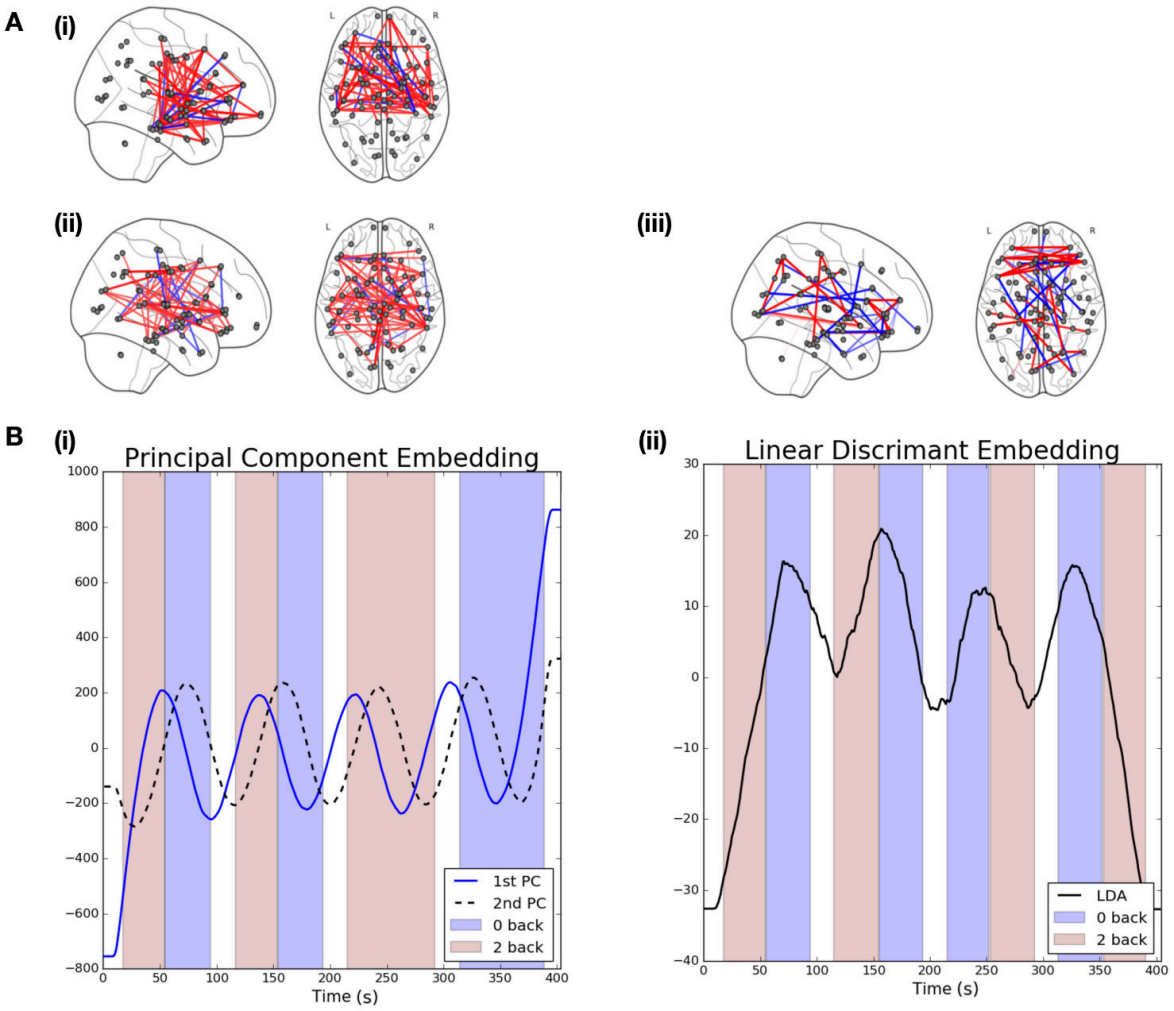
**Visualization of results when linear graph embedding methods are applied to HCP data. (A)** The brain networks visualize the functional connectivity networks associated with each of the embeddings. Positive edges are displayed in red while negative edges are visualized in blue. The networks shown correspond to the following embeddings: (i) 1st principal component embedding, (ii) 2nd principal component embedding, and (iii) the LDA-driven embedding. **(B)** Visualizations are provided for the PCA (left) and LDA (right) driven embeddings. The shaded background regions indicate the underlying cognitive task (blue indicates a 0-back task while red indicates a 2-back working memory task).

Figure 4B(i) shows the mean PCA-driven embeddings across all *S* = 206 subjects^2^. The background is colored to denote the task taking place at each point in time: red is used to denote 2-back working memory task while purple denotes a 0-back working memory task and a white background is indicative of rest. The embeddings associated with the first and second leading principal components display a clear oscillatory pattern which is correlated with the underlying task. Moreover, there is a lag in the oscillations of the 2nd principal component embedding with respect to the first, suggesting that distinct dynamics in the connectivity structure may be captured by each.

The left panel of Figure 4A shows the functional connectivity networks associated with each of the two principal component embeddings. Red edges indicate positive associations while blue edges indicate the opposite. The associated functional connectivity networks appear to reflect independent network dynamics. The network associated with the first principal component displays strong interhemispheric coupling, especially across motor regions but also for other mid-range connections, such as between motor and frontal regions as well as between frontal and medial temporal regions. Decreased inter-hemispheric coherence has previously been linked to poor working working memory performance in patients with traumatic brain injury (Kumar et al., 2009). In addition, interactions between the medial temporal lobe and frontal areas has been demonstrated for working memory tasks (Axmacher et al., 2008). On the other hand, the network associated with the second principal appears to show increased long-range coupling between frontal and parietal regions in the brain. This is in-line with the well-established engagement of the frontoparietal attention network during working memory tasks (Vossel et al., 2014; Wallis et al., 2015; Constantinidis and Klingberg, 2016). Finally, we note that the ordering of the embeddings is itself significant. In the context of resting state data we would expect the leading embedding to correspond to the Default Mode Network (DMN) as such a network has been widely reported to be active during rest (Greicius et al., 2009). However, from Figure 4A we note this is not the case, suggesting that the networks recovered are induced by the associated task.

In contrast to the PCA-driven embeddings, the LDA-driven embeddings are a supervised method which seeks to identify a reduced subset of edges which are discriminative across tasks. In this section we study the contrast between 0-back and 2-back working memory tasks^3^. As noted previously, two datasets were available for each subject. In such a supervised learning task care was taken to differentiate between the LR and RL acquisition datasets as there were small differences in task-design. The approach taken here was to build an LDA-driven embedding using only the LR acquisition datasets across all subjects and then validate this model using the unseen RL acquisition datasets. All (p2) potential edges were screened as described previously and only those selected over 60% of the time were studied. A threshold of 60% was selected in order to obtain embeddings with high discriminative accuracy whilst remaining easily interpretable. In particular, the variable screening strategy with a threshold of ρ = 60% served to reduce the number of candidate edges to p′=126<< (p2).

The results for the LDA-driven embedding are shown in Figure 4B(ii). This corresponds to the results of applying the LDA-driven embedding to the unseen RL acquisitions, averaged across all *S* subjects. The resulting embedding is strongly correlated with the onset of the 0-back working memory task (denoted by purple shading in the figure). This serves as an empirical validation that the embedding is able to discriminate across the two classes. The discriminative performance of the LDA-driven embedding was subsequently studied on a subject by subject basis by calculating the AUC score over the unseen RL acquisition dataset. The mean AUC score across all subjects was 0.69 with a standard deviation of 0.14.

We are also able to study the embedding in the context of the associated functional connectivity network, shown in Figure 4A(iii). While the networks associated with the PCA-driven embedding recovered edges which displayed high variability, the edges reported by the LDA-driven embedding are discriminative across the 0-back and 2-back working memory tasks.

## 5. Discussion

The study of dynamic functional connectivity networks is an important avenue of neuroscientific research which has become popular in recent years (Calhoun et al., 2014). As a result, many methodologies have been proposed through which to estimate time-varying connectivity networks. However, one aspect that has been overlooked has been how to effectively interpret and visualize the estimated networks and how these are modulated by the underlying task. In the past this issue has been partially addressed via the use of a wide range of methods including univariate testing on edges (Monti et al., 2014, 2015), tracking of graph metrics such as degree centrality (Calhoun et al., 2014) and clustering methods (Allen et al., 2012).

In this work we look to address these issues via the use of graph embedding methods based on linear projections over the set of edges. The motivation behind the use of linear methods stems from the fact that they may subsequently be interpreted in the context of functional connectivity. As a result, such methods allow for the identification of entire networks which vary throughout a task. In this manner, we are able obtain a more holistic understanding of the dynamic reconfigurations which are taking place.

Formally, the two embedding methods presented in this work are based on principal component analysis and linear discriminant analysis, respectively. These two approaches correspond to unsupervised and supervised learning methods, respectively, and can therefore be seen as complementary tools through which to understanding dynamic functional connectivity in further detail. The PCA-driven embedding presented is closely related to the eigen-connectivity approach introduced by Leonardi et al. (2013). Here PCA is employed to report a weighted combination of edges which demonstrates the largest variability over time. In the context of task-based fMRI, we hypothesize such edges will be related to the underlying task, however such an approach can also be applied in the context of resting-state data, indeed this is the original application presented by Leonardi et al. (2013). Conversely, the LDA-driven embedding corresponds to a supervised embedding algorithm which is explicitly designed for task-based fMRI data. First, a screening procedure is applied in order to weed out non-informative edges and yield sparse and interpretable networks. LDA is subsequently employed to learn an embedding which is discriminative across tasks.

The empirical capabilities of the proposed embeddings are studied throughout a series of simulation studies. These involved the generation of synthetic data whose properties resemble many of those typically reported in fMRI data. These simulations provide an important insight into the performance of the proposed graph embeddings. In particular, they serve to highlight the drop in performance as the number of regions increases given a fixed number of observations as can be clearly seen in Table 1. It is important to note that the aforementioned simulation study focused only on quantifying performance between two binary states. While this allows for the performance of the embeddings to be readily quantified, it follows that the dynamics of functional connectivity networks may by vary constantly and therefore be far more difficult to track.

We further note that the simulations presented have employed the SINGLE algorithm to obtain sparse functional connectivity networks. However, the graph embedding methods presented do not require sparse networks *per se* and may be employed in the context of dense networks as well. Moreover, while the simulations have focused primarily on the properties of the leading PCA or LDA embeddings, higher order components could be employed to study more complex dynamics in connectivity structure. Furthermore, studying the contributions of higher order components may potentially serve as a way of quantifying the complexity of the data at hand.

It is also important to consider the limitations of the proposed methods. Firstly, both embeddings are rooted on the assumption that an equal number of observations are available across all subjects. If this were not the case the estimated embeddings would be biased toward subjects with larger number of observations. Furthermore, the PCA-driven embedding described in Section 2.2 is also premised on the assumption that variability in connectivity structure over time dominates an inter-subject variability. It follows that if this were not to be the case, the embedding would instead recover the set of edges which display the greatest inter-subject variability. This is perhaps a more pertinent issue in the context of resting state data as we expect there to be clear changes in network structure induced by distinct cognitive tasks.

An important area for future work would be to study the performance of the proposed graph embedding methods on fMRI data consisting of very large numbers of brain regions (e.g., in the hundreds or thousands). It would also be of interest to consider more complex graph embeddings which further exploit the properties of networks, for example via the use of heat kernels (Chung et al., 2016a,b). Moreover, graph embedding methods could be employed in a variety of contexts. An exciting potential application is in personalized neurofeedback based on functional connectivity (Lorenz et al., 2015, 2016a,b, 2017; Monti et al., 2016). In such a setting, the use of graph embedding methods could potentially be employed to provide easily interpretable score for subjects to optimize via the use of neurofeedback.

## Author contributions

RM: Conceived methods. Ran simulations. Analyzed application results. Wrote the manuscript. RLo: Analyzed application results. Wrote the manuscript. PH: Analyzed application results. Wrote the manuscript. RLe: Analyzed application results. Wrote the manuscript. CA: Conceived methods. Wrote the manuscript. GM: Conceived methods. Wrote the manuscript.

### Conflict of interest statement

The authors declare that the research was conducted in the absence of any commercial or financial relationships that could be construed as a potential conflict of interest.

## Footnotes

^1^The selection of 206 subjects was based on the data available at the time of the study. Due to the computational burden associated with preprocessing and preparing data, only data for 206 randomly selected subjects was available.

^2^Note that only LR acquisition datasets plotted here, as the task design varied from LR to RL acquisitions.

^3^We note that alternative contrasts may also have been employed.
