# Supplementary material for: Decoding Time-Varying Functional Connectivity Networks via Linear Graph Embedding Methods
Source: Front Comput Neurosci. 2017 Mar 20;11:14. doi: 10.3389/fncom.2017.00014 (PMC5357637; doi:10.3389/fncom.2017.00014)
Supplement: Supplementary file 1 [file DataSheet1.pdf]

A Smooth Incremental Graphical Lasso Estimation algorithm

In this appendix we briefly review the SINGLE algorithm, through which to infer dynamic functional connectivity networks. The objective of the SINGLE algorithm is to infer a network at each observation by recovering the sparse conditional dependence structure. This corresponds to recovering the sparse support for the inverse covariance (precision) matrix. As such, for the sth subject we estimate a sequence of precision matrices $\{\Theta_i^{(s)}\} = \{\Theta_1^{(s)}, \ldots, \Theta_n^{(s)}\}$, where $\Theta_i^{(s)} \in \mathbb{R}^{p \times p}$ encodes the partial correlation structure at the ith observation for subject s.

In order to accurately recover the underlying connectivity structure, the SINGLE algorithm introduces both sparsity and temporal homogeneity constraints. Sparsity is introduced in order to ensure the estimation problem was well-posed as well as to remove spurious edges introduced by noise. Meanwhile, the introduction of temporal homogeneity is motivated by a desire to ensure changes in functional connectivity are only reported when strongly corroborated by evidence in the data. The SINGLE algorithm therefore seeks to find a balance between adequately describing the observed data while satisfying the aforementioned constraints. This is achieved by minimizing the following convex objective:

$$\{\hat{\Theta}_i^{(s)}\} = \underset{\Theta_i^{(s)}}{\operatorname{argmin}} \left\{ f(\{\Theta_i^{(s)}\}) + g_{\lambda_1, \lambda_2}(\{\Theta_i^{(s)}\}) \right\}. \tag{8}$$

Here $f(\{\Theta_i^{(s)}\}) = \sum_{i=1}^{T} -\log \det \Theta_i^{(s)} + \text{trace}\ (\hat{\Sigma}_i^{(s)} \Theta_i^{(s)})$ is proportional to the sum of negative log-likelihoods where $\hat{\Sigma}_i^{(s)}$ is the estimated covariance at time i. The penalty terms are enforced by the second term:

$$g_{\lambda_1, \lambda_2}(\{\Theta_i^{(s)}\}) = \lambda_1 \sum_{i=1}^{T} ||\Theta_i^{(s)}||_1 + \lambda_2 \sum_{i=2}^{T} ||\Theta_i^{(s)} - \Theta_{i-1}^{(s)}||_1.$$

Regularization parameters λ_1 and λ_2 each determine the extent of sparsity and temporal homogeneity respectively and can be tuned in by minimizing AIC.

B List of regions and associated MNI coordinates employed in HCP task

The table below details the regions employed. For each region, the MNI coordinates corresponding to the center of gravity are presented.

Table 2: Regions and MNI coordinates for HCP working memory task data

	Region	MNI coordinates		
1	Left thalamus	-11.40	-18.40	6.40
2	Left caudate	-14.60	9.20	9.20
3	Left putamen	-25.80	1.00	-0.80
4	Left pallidum	-20.20	-4.00	-0.80
5	Left hippocampus	-25.20	-23.60	-13.00
6	Left amygdala	-23.80	-6.00	-18.80
7	Left accumbens	-9.40	12.60	-7.00
8	Left ventral diencephalon	-9.00	-9.00	-9.00
9	Right thalamus	13.00	-17.80	7.00
10	Right caudate	16.20	9.00	10.00
11	Right putamen	26.40	1.60	-1.00
12	Right pallidum	21.80	-3.80	-0.40
13	Right hippocampus	26.80	-22.20	-12.80
14	Right amygdala	24.40	-4.40	-18.80
15	Right accumbens	9.60	12.20	-7.00
16	Right ventral diencephalon	-9.00	-9.00	-9.00
17	Left banks of the superior temporal sulcus	-54.80	-44.20	7.00
18	Left anterior cingulate	-4.80	21.00	27.00
19	Left middle frontal	-35.20	11.80	45.00
20	Left cuneus	-7.00	-78.60	19.60
21	Left entorhinal	-24.40	-4.80	-32.60
22	Left fusiform	-34.40	-42.60	-18.80
23	Left inferior parietal	-39.40	-68.00	29.60
24	Left inferior temporal	-49.00	-30.80	-22.80
25	Left isthmus of cingulate gyrus	-7.60	-45.20	18.80
26	Left lateral occipital	-28.60	-86.80	1.20
27	Left lateral orbitofrontal	-23.80	33.60	-15.00
28	Left lingual gyrus	-14.00	-67.20	-3.00
29	Left medial orbitofrontal	-7.00	40.40	-14.60
30	Left middle temporal	-56.20	-27.00	-12.20
31	Left parahippocampus	-24.20	-29.80	-17.80
32	Left paracentral	-7.80	-28.20	58.60
33	Left parsopercularis	-45.20	15.40	14.20
34	Left parsorbitalis	-40.80	41.40	-10.00
35	Left parstriangularis	-43.00	31.60	4.00
36	Left pericalcarine	-11.20	-79.80	7.40
37	Left postcentral	-42.00	-22.80	44.00
38	Left postior cingulate	-5.80	-17.80	38.80
39	Left precentral	-38.20	-9.40	43.20

Table 2: Regions and MNI coordinates for HCP working memory task data

	Region	MNI coordinates		
40	Left precuneus	-9.80	-57.00	37.20
41	Left rostral anterior cingulate	-5.40	37.20	3.60
42	Left rostral middle	-30.40	45.80	14.60
43	Left superior frontal	-11.40	28.60	41.80
44	Left superior parietal	-20.80	-63.80	47.20
45	Left superior temporal	-51.60	-12.80	-4.20
46	Left supramarginal	-51.00	-38.00	31.40
47	Left frontal pole	-8.80	64.60	-9.00
48	Left temporal pole	-32.40	12.20	-34.80
49	Left transverse temporal gyrus	-44.00	-22.00	8.00
50	Left insula	-35.20	-0.40	2.00
51	Right banks of the superior temporal sulcus	54.80	-39.40	6.40
52	Right anterior cingulate	6.60	22.20	27.20
53	Right middle frontal	36.20	13.00	45.20
54	Right cuneus	8.80	-77.80	20.80
55	Right entorhinal	24.40	-3.80	-32.00
56	Right fusiform	34.80	-39.20	-19.80
57	Right inferior parietal	43.40	-62.40	29.60
58	Right inferior temporal	49.20	-26.40	-24.80
59	Right isthmus of cingulate gyrus	9.20	-45.00	18.40
60	Right lateral occipital	31.40	-84.80	1.40
61	Right lateral orbitofrontal	23.20	32.40	-15.80
62	Right lingual gyrus	15.20	-66.20	-2.60
63	Right medial orbito frontal	6.80	39.80	-13.80
64	Right middle temporal	56.60	-22.40	-14.00
65	Right parahippocampus	25.40	-28.60	-17.40
66	Right paracentral	8.80	-26.20	57.60
67	Right parsopercularis	46.20	15.80	13.60
68	Right parsorbitalis	41.60	42.00	-10.20
69	Right parstriangularis	45.60	31.20	5.00
70	Right pericalcarine	12.40	-78.20	8.00
71	Right postcentral	42.40	-21.00	44.40
72	Right posterior cingulate	7.20	-17.20	38.80
73	Right precentral	39.00	-7.80	43.20
74	Right precuneus	11.40	-56.40	37.00
75	Right rostral anterior cingulate	6.60	37.60	5.00
76	Right rostral middle frontal	31.80	46.80	14.40
77	Right superior fron'tal	12.80	30.20	41.20
78	Right superior parietal	22.00	-63.00	48.40
79	Right superior temporal	52.40	-9.00	-5.40
80	Right supramarginal	52.40	-31.40	31.20
81	Right frontal pole	9.60	64.00	-10.00
82	Right temporal pole	33.00	14.00	-33.60
83	Right transverse temporal gyrus	45.00	-19.60	8.20
84	Right insula	36.20	1.00	0.60
